# A Kamm’s Circle-Based Potential Risk Estimation Scheme in the Local Dynamic Map Computation Enhanced by Binary Decision Diagrams

**DOI:** 10.3390/s22197253

**Published:** 2022-09-24

**Authors:** Arvind Kumar, Hiroaki Wagatsuma

**Affiliations:** Graduate School of Life Science and Systems Engineering, Kyushu Institute of Technology (Kyutech), 2-4 Hibikino, Wakamatsu-Ku, Kitakyushu 808-0196, Japan

**Keywords:** local dynamic map, binary decision diagrams, branching programs, Geohash, cooperative ITS, collision avoidance, Kamm’s/traction circle

## Abstract

Autonomous vehicles (AV) are a hot topic for safe mobility, which inevitably requires sensors to achieve autonomy, but relying too heavily on sensors will be a risk factor. A high-definition map (HD map) reduces the risk by giving geographical information if it covers dynamic information from moving entities on the road. Cooperative intelligent transport systems (C-ITS) are a prominent approach to solving the issue and local dynamic maps (LDMs) are expected to realize the ideal C-ITS. An actual LDM implementation requires a fine database design to be able to update the information to represent potential risks based on future interactions of vehicles. In the present study, we proposed an advanced method for embedding the geographical future occupancy of vehicles into the database by using a binary decision diagram (BDD). In our method, the geographical future occupancy of vehicles was formulated with Kamm’s circle. In computer experiments, sharing BDD-based occupancy data was successfully demonstrated in the ROS-based simulator with the linked list-based BDD. Algebraic operations in exchanged BDDs effectively managed future interactions such as data insertion and timing of collision avoidance in the LDM. This result opened a new door for the realization of the ideal LDM for safety in AVs.

## 1. Introduction

Driving a vehicle is an unavoidable daily task for millions of people, and, simultaneously, driving carries risks as a fate due to unsafe driving [1]. In the current direction toward autonomous vehicles (AVs) to contribute to a significant reduction of car accidents, the Society of Automotive Engineers (SAE) has defined various levels of autonomy from Level 0 (fully manual) to Level 5 (fully autonomous) [2]. Even though an autonomous ego vehicle can sense its environment effectively using sensors to keep safe navigation, physical sensors have limitations to sense behind buildings and obstacles [3]. In the sensing limitations, cooperative intelligent transport systems (C-ITS) are necessary to compensate for the drawbacks of sensors. In the proximity range between vulnerable road users and vehicles, sensors are necessary to detect what happens in front of vehicles at a high speed and high accuracy, while its accuracy gets worse in proportion to the distance. Therefore, sharing information among traffic participants represented in a consistent geographical way is crucial for safe mobility in middle and long distances. In a C-ITS, the mission of a high level of autonomy for safe driving has proposed for vehicles to use exchange information using vehicle to vehicle (V2V), vehicle to infrastructure (V2I), and vehicle to everything (V2X). Thus, a hieratical representation of static and dynamic information with different update time scales is critical to realize the facilities layer of every intelligent transport system (ITS) station. In other words, information from other participants in traffic conditions is aggregated in a consistent geometrical way. According to the demand, the SAFESPOT [4] project has introduced the concept of LDM, which integrates static road geometry with dynamic information from other vehicles and participants. In LDM, static, quasi-static, quasi-dynamic, and highly dynamic data are mapped consistently and promptly for real traffic scenarios. It indicated the integration of traffic participants’ information based on its dynamic properties to manage complex traffic scenarios. LDM is divided into four conceptual layers:

- Layer 1: Contains permanent static information. It is a map database that preferably contains detailed road map information with application to advanced driver assistance systems (ADAS).
- Layer 2: This layer is an extension of layer 1. It includes quasi-static information, e.g., traffic signs, trees, and buildings.
- Layer 3: LDM stores temporary information for a particular region in this layer, e.g., traffic jams, weather conditions, and traffic signals.
- Layer 4: Contains temporary information about dynamic or highly dynamic objects, e.g., moving vehicles and pedestrians.

In the realization, LDM requires relational, graph, or streaming databases [4,5,6,7] and assumed query languages for the possible database to store and monitor target data for the ITS station to handle various dynamic entities in the traffic scenarios mapped on the world model. In past studies of LDM implementations [5,6,7], the geographical occupancy of vehicles was handled in individual time segments, and risks due to collision of nearby vehicles were evaluated for those mutual distances in each time segment. It is a natural consideration in ordinary discrete time step models of moving objects. There is no problem if the occupancy is assured to be updated in the database with enough speed and precision to prevent accidents. However, assurance of updating speed and precision is seemingly unsolvable because increasing time resolution in the discrete-time model to adapt to high-speed movements, such as on highway roads, will be a trade-off issue with respect to computational costs. This is the reason why current implementations of LDM have limitations and inevitable drawbacks. Therefore, the issue that needs to be addressed in the LDM implementation is the possibility of extended representation of the geographical occupancy of vehicles across time segments or space-time representation over time. In this sense, rich space-time representation is vital for detecting potential future interactions and the safe navigation of vehicles.

Kumar et al. [8] have proposed a computational scheme with BDD encoding Geohash information. An extended present-and-future spatial representation of the LDM is required to tackle the above problem. A consistent framework can be established if BDD representations can be applied to reachable Geohash locations depending on each moving vehicle over time.

Geohash offers an efficient partitioning of geographical locations, and therefore it matches a Boolean string manipulation to treat geographical problems. The advantage of Boolean encoding associated with target Geohashes is set operations to minimize computational costs by implementing them into BDDs. It implies that the present-and-future spatial representation can be treated as a reachable set for the given vehicle over time by introducing the formulation of their future positions based on the concept of Kamm’s circle. The extended framework enables us to verify situations with risks of a collision with other cars in the form of algebraic operations in BDDs. In the present study, the following main contributions are discussed, as listed below:

1. The vehicle’s future geographical occupancy over time as a feature in the LDM.
2. A extended method of data representation for a vehicle’s geographical occupancy information using a BDD.
3. Possible algebraic operations between the exchanged BDDs can confirm the possibility of future interaction, which is consistent with the C-ITS nature of data sharing.
4. Ways of data insertion and database operations for vehicle properties in the linked-list-based BDD running on the PostgreSQL database-based LDM.

The rest of the paper is structured as follows: Section 2 reviews the state of the art of the LDM approach. Section 3 describes the materials and methods used in the present study. Section 4 and Section 5 describe the experimental setup and analyzed the results. Finally, Section 6 examines the discussion and future work and concludes the paper.

## 2. Literature Review

The SAFESPOT project was co-funded by the European Commission Information Society and Media and was supported by European Council for Automotive R&D (EUCAR) from 2006 to 2010 and introduced the LDM [4] to manage the data in the C-ITS scenario, which constructed an LDM on the top of a database. It indicated that the LDM integrates information received from the vehicles and infrastructure with geometry information is realized in the database. In other words, it provides real-time data of static, temporary, and dynamic elements involved in a traffic scenario. Tele Atlas (PG-LDM) and NAVTEQ (NAVTEQ-LDM) were two implementations of the system. PG-LDM introduced a PostgreSQL database with a PostGIS extension, whereas NAVTEQ-LDM introduced SQLite. A schema for the relational databases for constructing the LDM is also crucial for an actual application. In their system, grouped tables in four layers are updated depending on the dynamic properties of the target, moving entities, and their relationships with data stored in other tables. Thus, tables were divided into four conceptual categories or layers which are consistent with the LDM concept.

The European Telecommunications Standards Institute (ETSI) and International Organization for Standardization (ISO) have made standardization efforts for LDM. The initial standard was given by ETSI as TR 102 863 (V1.1.1) [9], which described LDM as an embedded conceptual data storage in an ITS station. It maintains the topographical, positional, and status information related to the ITS station within the host station’s geographic area. Therefore, it identified LDM as a key facility function in the facilities layer of an ITS station. Essential data sources of LDM were discussed in cooperative awareness messages (CAMs) and decentralized environmental notification messages (DENMs). For standardization, various applications of the LDM were considered such as “Cooperative navigation Location-based services", which can provide location-based information for cooperative navigation. In the ITS applications analysis, the functionality [9] portion of the standard clearly mentioned use cases related to the LDM, for example, UC_CA_03 (across traffic turn collision risk warning), UC_CA_04 (merging traffic turn collision risk warning), UC_CA_05 (co-operative merging assistance), UC_CA_06 (intersection collision warning), and UC_CA_07 (co-operative forward collision warning) use cases. The document highlighted the requirement of a mechanism to update the LDM by storing processed information on target moving entities and stressed the importance of the LDM for other applications and stakeholders in transportation. It indicates that the selection of the updating method in the database is not a minor problem at the implementation level because computational costs for estimating vehicle interactions increase rapidly in principle when the number of vehicles increases. Therefore, the BDD-based geographical information storing was proposed by Kumar et al. [8], which is easily extended to Geohash locations as a reachable set to represent future locations, allowing the database to store those in JSON data format. According to the standard, various types of data were termed Type 1 (static), Type 2 (transient static), Type 3 (transient dynamic), and Type 4 (highly dynamic). Especially in the case of the highly dynamic data (Type 4) focusing on nearby vehicles, it requests an extension of the LDM to support the “vehicle occupancy” as discussed above. Indeed, ETSI EN 302 895 (V1.1.0) [10] extended the previous report and added descriptions of new functionalities associated with compositional data structures and LDM data providers and customers. In an international standard, ISO/TS 17931:2013 [11] and ISO/TS 18750:2015 [12] defined a comparable standard to ETSI.

In past studies on LDM implementations, Eggert et al. [5] proposed relational local dynamic maps (R-LDM), a fully interconnected graph-based approach, instead of layered structures. They were concerned with computational costs due to the complexity of layered models to update data and proposed a consistent world model as an improvement, which used the Neo4j database and CYPHER query language for the LDM implementation. Camera-to-map alignment and risk-based behavior were demonstrated in their model to exhibit the benefit of a fully interconnected graph-based approach, while inconsistency with layered data structures in standards discussed above is a concern for LDM data providers and customers. Eiter et al. [7] introduced semantic web technologies and combined ontologies with a spatial stream database. LDM ontology with expressive spatial-stream query language is beneficial and suitable for the hierarchical data structure to infer new information over streams. They demonstrated the integration of semantic web technologies with LDM and V2X. The experiment involved the PostgreSQL extension PIPELINEDB database and PTV VISSIM simulation environment, which may have a potential problem of computational costs due to limitations of processing speed in reasoner accompanied with combined ontologies. Netten et al. [13] introduced DynaMap, a dynamic map for roadside or central ITS stations. They discussed the importance of the difference between the dynamic map requirement for roadside units and the dynamic map for vehicles and formulated an architecture for world models, objects, and data sinks. Koenders et al. [14] focused on the fact that conventional LDM implementations cannot store the data of all things simultaneously and improved it with a streamed filtering technique to delete irrelevant data. Their relational schema was a sophisticated model to carry tables for areas, roads, and objects. Zoghby et al. [15] built a distributed LDM in the context of VANets (vehicular ad hoc networks). In their model, vehicles cooperate to increase their field of view and provide an extended map called a dynamic public map (DPM), depending on the dynamic properties of moving entities represented in dynamic distributed maps (DDMs). Their computer experiments demonstrated that a number of vehicles can be treated in the distributed dynamic map consistently. Nieto et al. [16] implemented the real-time LDM using RTMaps as a middleware. As a successful fusion, the database was designed in the vehicle and roadside units (RSU). Biral et al. [17] described the SAFESTRIP project, and their developed road strips can detect and estimate lateral and longitudinal positions of the detected vehicle at the lane level.

The LDM concept was extended not only to road vehicles but also to aerial vehicles. Lee et al. [18] proposed an algorithm to generate a 3D local dynamic map for unmanned aerial vehicles (UAVs). García et al. [19] recently introduced an interoperable graph-based LDM (iLDM) using Neo4j. In their model, the system introduced a graph database for the LDM construction, the same as R-LDM proposed by Eggert et al. [5]. In iLDM, a common input system is provided an interoperable data access across multiple data sources, which is OpenLABEL as a common data format. Shimada et al. [6] implemented the LDM that was assumed to be fully compatible with the specification of the SAFESPOT project and demonstrated the performance of the LDM while changing the number of vehicles in their computer environments for the collision detection task. In this case, LDM was established with the Postgres database with PostGIS extension and a geographical map in the database with the “osm2pgsql” tool for data in static layers concerning tables. For sensor information in their computer experiments, PreScan and Simulink generated sensor data depending on vehicle movements by accessing dynamic layer tables.

## 3. Materials and Methods

This section briefly introduces the material, methods, and technologies to achieve our objective.

### 3.1. Geohash

**Figure 1:**
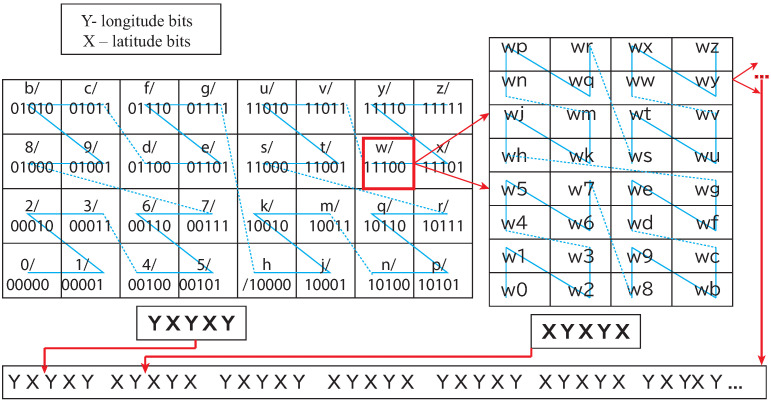
Geohash follows an alternate sequence of space-filling curves. Characters binary representation determining latitude X bits and longitude Y bits cross bit by bit.

In our proposed model, Geohash was introduced to represent the geographical locations, which is a sequence of characters consisting of English letters except “a”, “i”, “l”, “o”, and digits 0–9 at every level of the representation. The string length corresponds to the size of the geographical area designated by the Geohash, as shown in Table 1. It is a hierarchical spatial data structure subdividing the space into smaller subspaces depending on the Geohash length. For example, the first character divides the space into 4 × 8 (four rows and eight columns), and the division of regions alternates between 8 × 4 and 4 × 8 ([20,21]). A space-filling curve decides the sequence number of the areas. When alternate characters’ binary representations are combined in Geohash, two strings for determining row X (latitude bits) and column Y (longitude bits) cross bit by bit, see Figure 1.

**Figure 2:**
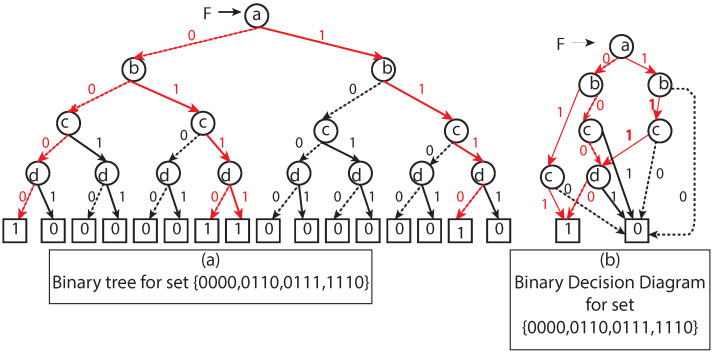
(**a**) Binary decision tree representation for a given set has a fixed size and is large compared to BDD representation. (**b**) Binary decision diagram representation for a given function has compact representation.

According to the above formulation, each Geohash has its unique binary representation. This binary representation for locating a region in space motivated us to use BDD since BDDs are relatively small for multiple Boolean functions compared to the corresponding binary tree representation (see Figure 2). It supports logical operations on BDDs, which correspond to equivalent set-theoretic operations (Section 3.3). Interestingly, computer-aided design (CAD), formal verification, and other related fields have successfully introduced BDDs for Boolean operations.

### 3.2. Boolean Function and Reduced Ordered Binary Decision Diagrams (ROBDD)

In the proposed model, ROBDD was introduced to represent the Boolean function represented by the set of Geohash locations.

#### 3.2.1. Boolean Function

A Boolean function is of the form $f:{\left\{0,1\right\}}^{k}\to \left\{0,1\right\}$, where k-tuples of Boolean variables takes values to 0 (false) or 1 (true). Suppose valuation *V* means the total combination of values that k-tuple Boolean variables can take. Each k-tuple assignment in *V* can be written as Γ:v→[0,1] from a value in fixed set V to a Boolean value, where vϵV. The Boolean function can also be represented using Boolean variables and Boolean operations (and,or,not), also known as literals, e.g., $x_{1}x_{2}\bar{x_{3}}+x_{4}$, where concatenation, + and $\bar{x}$ represent and, or, and not operations over variables, respectively.

#### 3.2.2. Reduced Ordered Binary Decision Diagrams (ROBDD)

The BDD is a graph representation of the Boolean functions. The basic idea behind the BDD is divide and conquer. More specifically, BDD is a rooted directed acyclic graph (DAG), where non-leaf nodes have labels with Boolean variables and leaf nodes have labels 0 (false) or 1 (true), which correspond to Boolean function output.

BDD can represent most of the Boolean functions in feasible size compared to the truth table or binary tree representation for Boolean functions that always take $2^{n}$ space. Sheldon B. Akers [22] first introduced the Boolean function in terms of a diagram. Later, Randal E. Bryant [23] introduced ROBDD (reduced ordered binary decision diagram), in which the relative ordering of variables on each path from the root to the leaf is fixed (also known as ordered binary decision diagram (OBDD)), and it combines the isomorphic subgraphs present in the graph to create ROBDD.

Each OBDD has the following components [24]—$G=(\left(Q,v_{0},E\right),V\cup \left\{0,1\right\},<,L)$:

- $\left(Q,v_{0},E\right)$ is a rooted directed acyclic graph. *Q* is a finite set of nodes. $v_{0}$ is the root node and E⊂Q×Q. Each non-leaf node has its successors, namely low and high.
- *V* is a finite set of Boolean variables.
- < is a total order on V∪{0,1}.
- *L* is a mapping satisfying the following conditions:
  – Leafs are mapped to 0 and 1 and non-leaf nodes are mapped to *V*.
  – If (v,v’) ∈E then L(v) < L(v’).

Graph *G* over Boolean variables *V* represents a Boolean function. The interpretation of BDD is based on the Shannon expansion.

(1) $$f=\bar{x}f\left[x\right]+xf\left[\bar{x}\right]$$

According to the Shannon expansion, each internal node of the graph has low and high, and ROBDD can be obtained from OBDD by minimizing the redundancy in the representation using the following rules:

- Merge all zero and one nodes to a single unit of zero and one node.
- Merge any isomorphic nodes, i.e., if l(x)=l(y) and h(x)=h(y) then merge these nodes into one and point all incoming nodes to any one of them. Here *l* and *h* represent the low and high child of any given node of a graph.
- Eliminate any node that has two children nodes as isomorphic.

The size of the ROBDD depends on the represented function and the variable order in the proposed model. For a given variable order, ROBDD representation for the Boolean function is the canonical representation, i.e., the function has a unique representation.

Due to the evolution of decision diagrams over the years, BDDs have many variants such as ROBDD, ZDD (zero suppressed decision diagram) [25], SBDD (shared binary decision diagram) [26], MTBDD (multi-terminal binary decision diagram) [27], and many more. In the present study, ROBDD was introduced as well as BDD. For simplicity, ROBDD is abbreviated as BDD in the following sections.

### 3.3. Geohash Set as a BDD

Geohash was introduced as a primary unit space, and then its size varied depending on the number of characters/levels in Geohash. The present study formulated a Geohash of ten levels/characters. It has approximately 0.59 m from north to south and 0.96 m from east to west, see Table 1. For the representation of collection events, Geohashes were encoded by using BDD.

**Figure 3:**
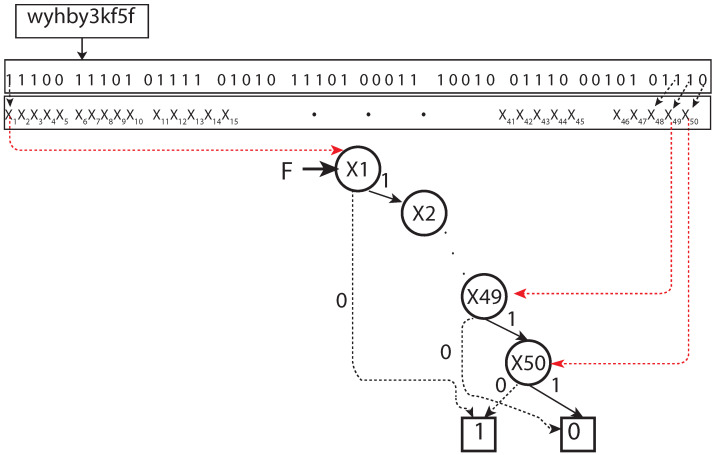
BDD representation for a unit Geohash.

**Figure 4:**
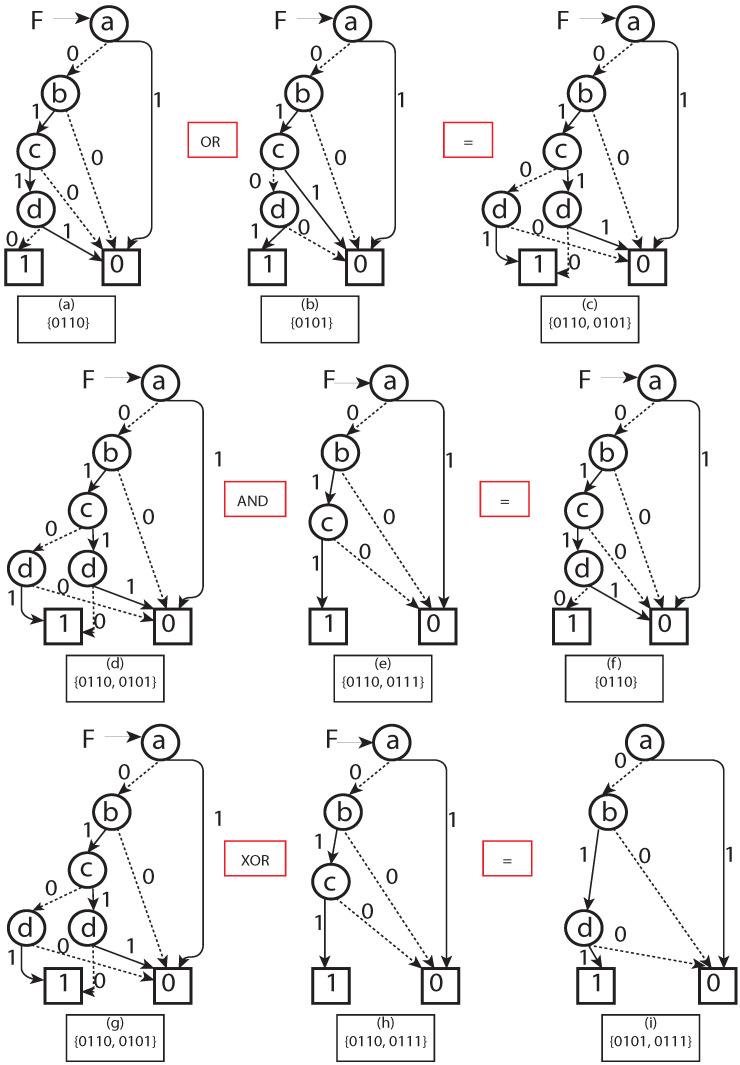
(**a**–**c**) BDD OR operation is equivalent to set union operation. (**d**–**f**) BDD AND operation is equivalent to set intersection operation. (**g**–**i**) BDD XOR operation is equivalent to a set symmetric difference operation.

**Figure 5:**
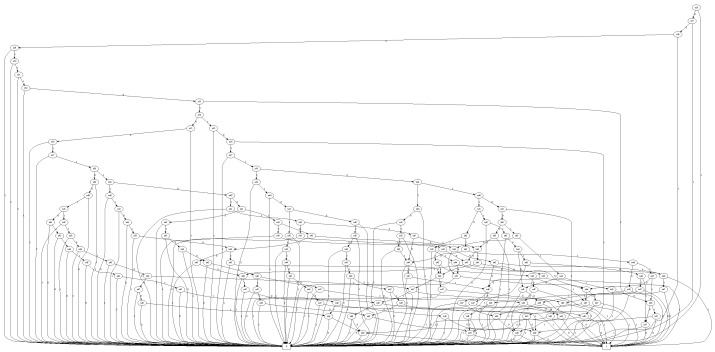
BDD for a set of 701 Geohashes. (Interconnection between the 25th–50th nodes is shown for brevity.)

1. *BDD representation of a unit Geohash:* A Geohash is a unique symbolic representation of all the points available within the given area on the earth. For each character in Geohash, 32 values (English letters except “a”, “i”, “l”, “o”, and decimal system digits 0–9) are possible to use, and, therefore, in our model, five Boolean variables ($2^{5}=32$) were applied correspondingly (Figure 1). Consequently, five nodes in a BDD were used to represent the corresponding Boolean variables for a binary representation of a given character in a Geohash. For a given Geohash, each character had five corresponding nodes in the BDD. For a Geohash of 10 characters/levels, 50 nodes were needed for corresponding bits, plus two extra nodes representing zero (false) and one (true) leaf node in a BDD. (for experiments, the vehicle was assumed to be within a Geohash, having a distance of 4872 m (north to south) and 3955 m (east to west); hence, a five-level BDD with 25 nodes served the purpose), i.e., the first five levels of Geohash did not change in our setting. Every corresponding node, low or high, has its values depending on the Boolean function represented. Therefore, to represent a single Geohash using BDD corresponding binary string ends at one (1) node of a BDD, and all other binary strings end in zero (0) (Figure 3).
2. *BDD representation of a set of Geohash:* A synthesis of BDD was applied (we borrowed the term “synthesis" from [28]) to represent a set of Geohashes in a single BDD. In the BDD synthesis, BDDs were built for complex sets/functions representing Geohash locations (e.g., BDD for function *f* can combine with function *g* to represent BDD for fANDg, fORg, NOTf, fXORg). Corresponding set interpretations were necessary for a given BDD representing *f* and *g* sets (here Geohash sets) of the above synthesis operations. The apply method in [23] was introduced to achieve the following operations:
  (a)  fORg is the set union operation. f∪g={α∣α∈forα∈g}
  (b)  fANDg is the set intersection operation. f∩g={α∣α∈fandα∈g}
  (c)  fXORg is the set symmetric difference operation. f⊕g=(f\g)∪(f\g)
  Accordingly, adding a Geohash in a given BDD representation of a set of Geohashes was performed with OR operation between two corresponding BDDs representations (Figure 4). For example, encoded BDD using the OR operation of a set of 701 Geohash BDDs is shown in Figure 5.

### 3.4. Reachable Positions by a Vehicle over Time t

For the prediction and inclusion of future occupancy of vehicles in the LDM, Kamm’s circles were introduced to formulate an abstraction of reach and reachable sets, as discussed in [29,30]. For example, reach and reachable sets in the discrete finite set of states are given as follows.

Let S=(X,U,T) be a finite state machine. Where *X* is the finite set of states, *U* is the finite set of control inputs, and T:X×U→X is the transition function. $X_{0}$ is the set of initial states.

#### Reach/Reachable Sets and Abstraction

**Figure 6:**
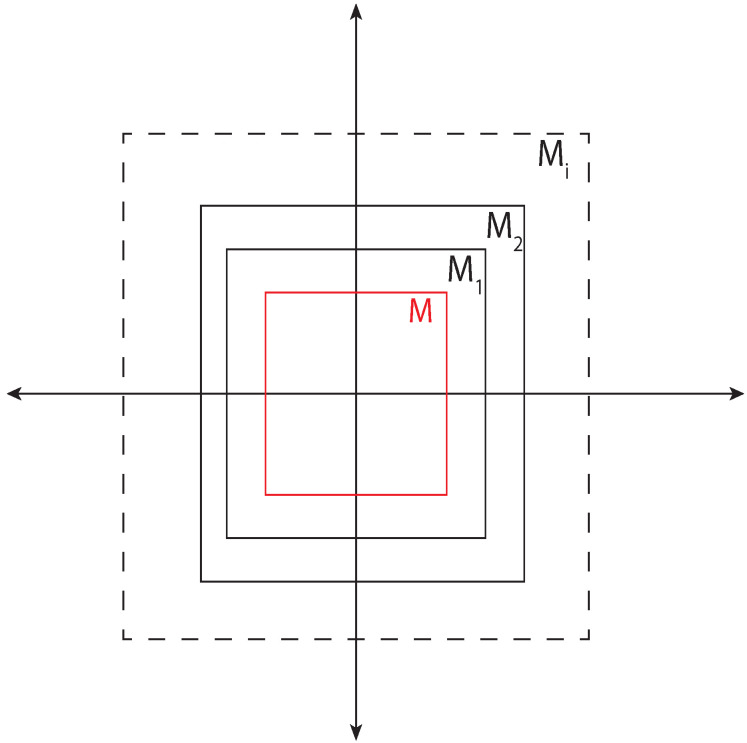
Abstraction of a model contains all reachable states which are reachable by the original model. Here states reachable by all abstraction models $M_{1},M_{2},\cdots ,M_{i}$ contain reachable states by a vehicle model *M*.

Reach Set—The set of states *x* at time *t* for which sequence of control inputs $u_{0},u_{1},\cdots ,u_{t-1}$ exists from the initial states $x_{0}\in X_{0}$ are known as reach set $R(X_{0},t)$ [31].
Reachable Set—Reachable set at time *t* is the union of all the reach sets ≤t
  (2) $$\bar{R}\left(X_{0},t\right)=\cup_{s\leq t}R\left(X_{0},t\right)$$
Abstraction—For a model (refer to definition in [29]) *M* of a given vehicle, abstraction was defined as the model $M_{i}$ if the reachable set of the abstraction contains the reachable set of the model *M* (Figure 6).

  (3) $$\forall t>0:R\left(M,t\right)\subseteq R\left(M_{i},t\right)$$

**Figure 7:**
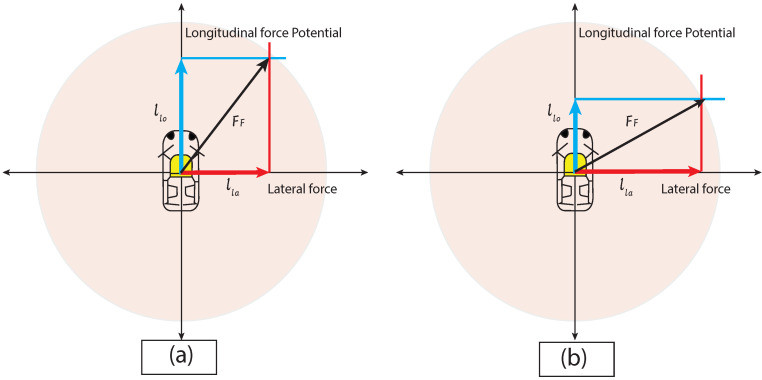
Overall force is limited to $F_{f}$. (**a**) An increase in longitudinal force limits the lateral force. (**b**) An increase in lateral force limits the longitudinal force.

Kamm’s circle was introduced as an abstraction [29] for the reachable positions of a given car. Kamm’s circle as abstraction overapproximates the locations to estimate vehicle positions that may be reached in a closed distance. Kamm’s/traction circle limits the maximum forces applicable between tires and the road. In the model, $a_{lo}$ longitudinal and $a_{la}$ lateral acceleration satisfies Equations 4 and 5 without losing the grip (Figure 7).

(4) $$a_{lo}^{2}+a_{la}^{2}⩽F_{f}$$

(5) $$a_{lo}^{2}+a_{la}^{2}⩽\mu_{r}^{2}g^{2}$$

where $\mu_{r}$ and *g* represent the friction coefficient and gravitational acceleration, respectively. Equation (5) forms and circle of radius $\frac{1}{2}\mu_{r}gt^{2}$ and using Equation (6) $a_{max}=\mu_{r}g$.

**Figure 8:**
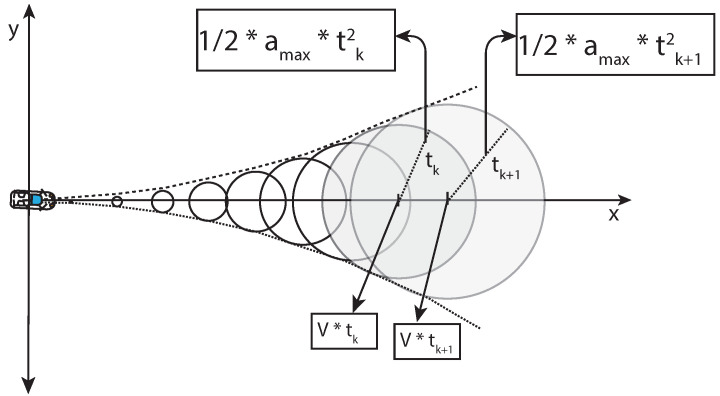
Kamm’s circle.

It is challenging to consider the trajectory of whether the representation of a vehicle over time is possible or not. Past studies [29,32] described the overapproximated occupancy at time t with center c(t) and radius r(t), as shown in Figure 8:

(6) $$c\left(t\right)=\left[\begin{array}{c} s_{x}\left(0\right)\\ s_{y}\left(0\right) \end{array}\right]+\left[\begin{array}{c} v_{x}\left(0\right)\\ v_{y}\left(0\right) \end{array}\right]\;\;;\;\;r\left(t\right)=\frac{1}{2}a_{max}t^{2}$$

where

- c(t) is a position of a vehicle at time t.
- $s_{x}\left(0\right)\;and\;s_{y}\left(0\right)$ is the position of the vehicle at time t = 0.
- $v_{x}\left(0\right)\;and\;v_{y}\left(0\right)$ is the velocities in the x and y directions of the vehicle at time t = 0.
- r(t) is the radius of a Kamm’s/traction circle at time t.
- $a_{max}$ is the maximum acceleration possible of a given vehicle.

$\frac{1}{2}a_{max}t_{k}^{2}$ is the radius of any Kamm’s circle at time $t_{k}$ (Figure 8). Kamm’s circle at time $t_{k}$ contributes to the reach set at that time. The union of such reach sets at time <=t gives the reachable set (reachable positions in our case). This formulation was applied to approximate the future places reachable by the vehicle and include the Geohash of the reachable Geohash locations as a BDD in the LDM.

### 3.5. BDD for Geohash Set Enclosing Kamm’s Circle

Following operations from [23], Geohash-based BDD manipulation was used in the present study.

1. *Reduce:* Give reduced BDD in its canonical form.
2. *Apply:* Perform synthesis operation between two BDDs. $f_{1}\;<op>f_{2}$.
3. *Satisfy-One:* Returns any one element in $S_{f}$, where $S_{f}$ is the set of all Geohash represented by a given BDD.
4. *Satisfy-All:* Output $S_{f}$. All Geohashes, a given BDD, satisfy.

The previous section discussed vehicle occupancy possible over time *t* using the Kamm’s/traction circle. This subsection explored the algorithms used to generate the BDD for such vehicle occupancy. Algorithm 1 was designed to find neighboring Geohash BDD for a given encoded BDD; Algorithm 2 was proposed to generate the concerned Kamm’s/traction circle BDD.
**Algorithm 1:** Algorithm to find neighboring Geohash BDD.1 Input: **inpGeo** -Geohash BDD. **h** in {west, east, null}, **v** in {south, north, null}.2 Output: Neighbour in east, west, north, south, north-west, north-east, south-east, south-west Geohash BDD.3 S = Satisfy-One(inpGeo)4 T = [1,1,1,1,1,1,1,1,1,1,1,1,1,1,1,1,1,1,1,1,1,1,1,1,1]5 for i = 0 to T.length:6    for j = 0 to i:7       If j%2==0 then:8          If h==west then:9            T[j] = T[j] and not(S[j])10          elif h==east then:11            T[j] = T[j] and S[j]12       else:13          If v==south then:14           T[j] = T[j] and not(S[j])15          elif v==north then:16            T[j] = T[j] and S[j]17 if h!=null:18    T[T.length-1] = 119 if v!=null:20    T[T.length-2] = 121 for i = 0 to T.length:22    S[i] = S[i] xor T[i]23 return createStringtoBDD(S)

In Algorithm 1, the Satisfy-One [23] method was introduced to find an input that satisfies the BDD. Then, T calculates the transition bits [33] needed to calculate the neighboring Geohash. For the completion of the operations, the bit string was generated for nearby vehicles after the XOR operation between the satisfying input with the transition string.

Subsequently, the mid-point circle generation algorithm is modified (see Algorithm 3) to find the BDD for all the Geohash present inside a given circle of radius *r*. The mid-point circle generation algorithm uses the eight-way symmetry present in the circle. If the points in one octant are possible to calculate, the points in all seven other octants can be generated. Assuming the center is (0,0), for mid-point circle generation algorithm in Step I, calculate the first square/pixel at $\left(x_{0},y_{0}\right)=\left(0,r\right)$. After that, a decision parameter *p* finds its use to generate the next squares/pixels in the first quadrant. Step II calculates the *p* decision parameter initial value $p_{0}=\frac{5}{4}-r$. Then, in Step III, depending on the weight of decision parameter *p*, the successive value of *p* and squares/pixels take their values as follows:

If $p_{k}<0$ then:
     $\left(x_{k},y_{k}\right)$ = $\left(x_{k}+1,y_{k}\right)$ and new $p_{k}$ is calculated as $p_{k+1}=p_{k}+2x_{k+1}+1$
else:
     $\left(x_{k},y_{k}\right)$ = $\left(x_{k}+1,y_{k}-1\right)$ and new $p_{k}$ is calculated as $p_{k+1}=p_{k}+2x_{k+1}+1-2y_{k+1}$

Next, in Step IV, the algorithm determines symmetry points in the other seven octants and repeats steps III to IV until x<=y.

**Figure 9:**
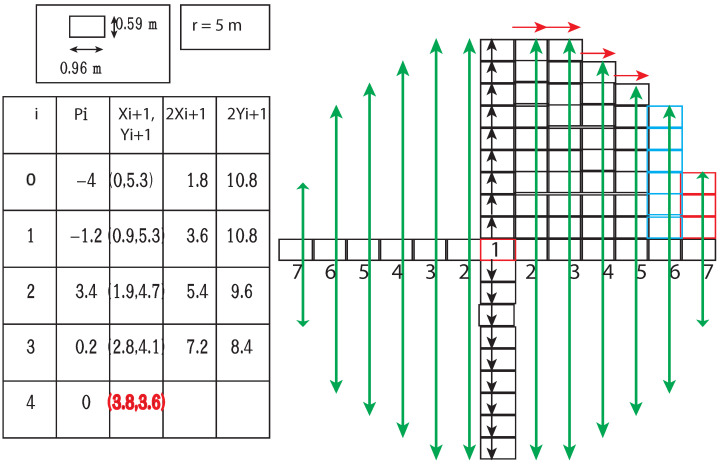
Modified midpoint circle algorithm.

The modified mid-point circle generation Algorithm 2 generated the BDD of all the Geohashes in the circle of radius *r*(Figure 9). Step I is the initialization step. In Step II, the BDD for all the Geohashes with black arrows were generated as shown in Figure 9 and merged with the BDD (circle_BDD) to represent all Geohashes within the circle by using or operation, as the or operation on BDD is the equivalent set union operation. Then, in Step III, the algorithm initialized the decision parameter *p* with p=INTEGER(ROUND(5/4)−r). Step IV, depending on the value of *p*, generated successive Geohashes available in the boundary of the first quadrant (along red arrows); successive *p* values and more parameters of the circle were as follows:

if p<=0:
  Generate east BDD and union it with circle_BDD. Additionally, update the value x_k=x_k+0.96, e_count=e_count+1 and record the north limit of this BDD from the center. Finally, update the value of the decision parameter as p=p+2∗x_k+1.
else:
  Generate southeast BDD and union it with circle_BDD. Additionally, update the value x_k=x_k+0.96; y_k=y_k−0.59 and record the north and east limit of this BDD from the center. Finally, update the value of the decision parameter as p=p+2∗x_k+1−2∗y_k.

After completion of Step IV, quad1_north_limit contained the distance (in no. of Geohash) of all Geohash in the first octant of the circle in the north direction and quad1_east_limit distance (in no. of Geohash) of Geohash in the first octant of the circle in the east direction with respect to inpGeo.

In Step V, the algorithm generated BDD3 and BDD4 in the east and west direction of the origin (Figure 9), and depending on the value of BDD3 (east) and BDD4 (west) Geohash, the algorithm generated BDDs in the north and south directions taken the first quadrant north limit as a limit (green arrow). All caused BDDs are taken in union with circle BDD (circle_BDD). Finally, in Step VI, depending on the value of the east limit of the first octant, new limit *a* is calculated and generated BDDs on added with BDD representing the circle.

  
**Algorithm 2:** Modified midpoint circle generation algorithm.1 Input: **inpGeo**—Center Geohash BDD, **r**—radius in meters unit.2 Output: BDD for a set of Geohashes enclosing Kamm’s circle.3 /*Step I.*/4 up_count = ⌈radius/0.59⌉5 quad1_north_limit = quad1_east_limit = []6 circle_BDD = inpGeo7 BDD1 = BDD2 = BDD3 = BDD4 = inpGeo8 x_k = y_k = 09 n_count = e_count = 010 /*Step II.*/11 for k = 0 to up_count:12    *BDD1 = Generate north BDD of BDDs.*13    *BDD2 = Generate south BDD of BDDs.*14    y_k = y_k + 0.5915    n_count = n_count + 116    BDD1∪BDD2∪circle_BDD./*Apply union with circle_BDD*/17 /*Step III.*/18 p = INT(ROUND(5/4) - r)19 /*Step IV.*/20 while x_k<=y_k:21    if p<=0:22       *BDD1 = Generate east BDD of BDD1.*23       x_k = x_k + 0.9624       e_count = e_count + 125       quad1_north_limit.append(n_count)26       BDD1∪circle_BDD.27       p = p + 2 * x_k + 128    else:29       *BDD1 = Generate south east BDD of BDD1.*30       BDD1∪circle_BDD.31       x_k = x_k + 0.9632       y_k = y_k - 0.5933       quad1_east_limit.append(e_count)34       e_count = e_count + 135       n_count = n_count - 136       quad1_north_limit.append(n_count)37       p = p + 2 * x_k + 1 - 2 * y_k38 quad1_east_limit.append(x_count)39 /*Step V.*/40 for w = 0 to quad1_east_limit.length-1:41    *Generate BDD3 and BDD4 east and west of BDD3 respectively.*42    *circle_BDD∪BDD3∪BDD4*43    for k = 0 to quad1_north_limit[w]:44       *Generate BDD5 and BDD6 north and south of BDD3 respectively.*45       *Generate BDD7 and BDD8 north and south of BDD4 respectively.*46       *circle_BDD∪BDD5∪BDD6∪BDD7∪BDD8*47 /*Step VI.*/48 for w = quad1_east_limit.length-1 to 0:49    *Generate BDD3 and BDD4 east and west of BDD3 respectively.*50    circle_BDD∪BDD3∪BDD451    a = ⌈x_count[w]∗(1.6)⌉ /*1.6, Geohash (10 level) breadth to height ratio*/52    if a>=quad1_north_limit[w] then:53       a = quad1_north_limit[w]54    for k = 0 to a:55       *Generate BDD5 and BDD6 north and south of BDD3 respectively.*56       *Generate BDD7 and BDD8 north and south of BDD4 respectively.*57       circle_BDD∪BDD5∪BDD6∪BDD7∪BDD858 return circle_BDD

**Algorithm 3:** Midpoint circle generation algorithm.
1 Input: **r**—radius of a circle, ($x_{c}$,$y_{c}$) center of the circle.
2 Output: Squares to include on a square grid to form a circle of radius r.
3 I. First square to include ($\left(x_{0},y_{0}\right)=\left(0,r\right)$)
4 II. Calculate the initial value for the decision parameter.
                                                                $p_{0}=\frac{5}{4}-r$
 III. For successive values of k, $\left(x_{k},y_{k}\right)$ is determined as follows.
 If $p_{k}<0$ then:
     $\left(x_{k},y_{k}\right)$ = $\left(x_{k}+1,y_{k}\right)$ and new $p_{k}$ is calculated as $p_{k+1}=p_{k}+2x_{k+1}+1$
 else:
     $\left(x_{k},y_{k}\right)$ = $\left(x_{k}+1,y_{k}-1\right)$ and new $p_{k}$ is calculated as
     $p_{k+1}=p_{k}+2x_{k+1}+1-2y_{k+1}$
 IV. Determine the symmetry points in the other seven octants.
 V. Repeat Steps III to IV until x≤y.


## 4. Experiment

**Figure 10:**
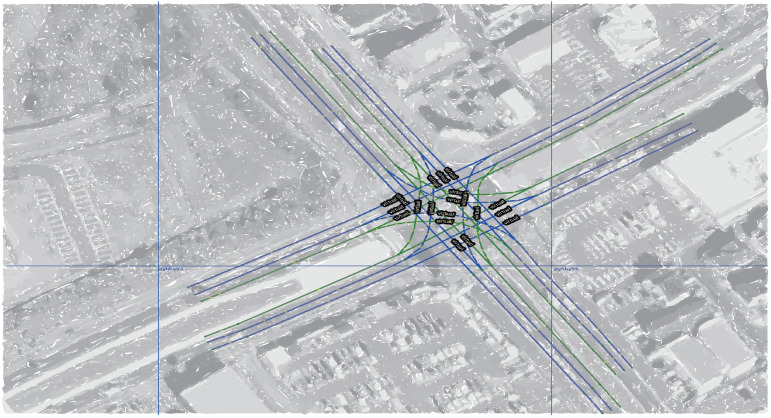
(Scenario-1) An example of an intersection center (geographical data from OSM [36] are illustrated as a superimposed background image).

**Figure 11:**
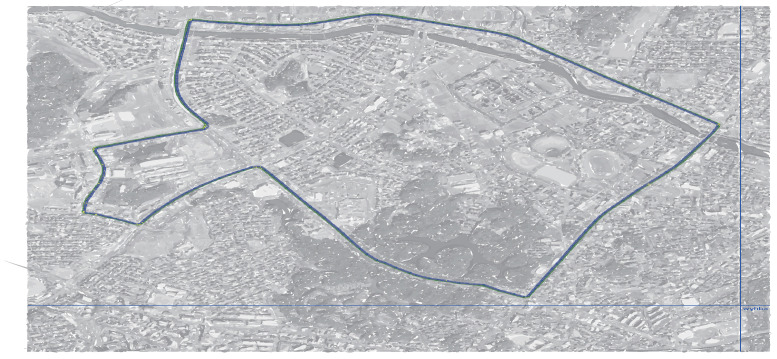
(Scenario-2) A city road scenario (geographical data from OSM [36] are illustrated as superimposed background image).

**Figure 12:**
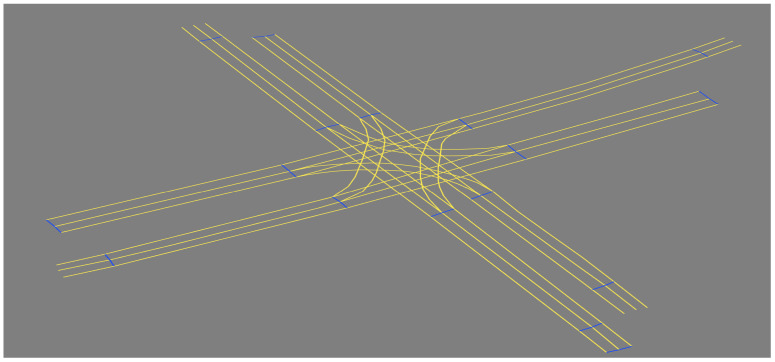
Loaded lanelet map in CoincarSIM simulator.

In computer experiments, the lanelet map [34] was introduced to simulate Scenario-1 and Scenario-2 (as shown in Figure 10 and Figure 11, respectively) using JavaOpenStreetMap (JOSM) and loaded them into ROS-based simulator CoInCar-Sim [35] with multiple vehicles. Figure 12 demonstrates the loaded lanelet map corresponding to Figure 10 in the above simulator. In addition, the vehicle data are generated in Scenario-2 and stored as a CSV file. Data are fed from CSV files into the LDM at every interval of 50 ms. The ego vehicle queries the LDM to get information for the collision detection task every 100 ms, the same as the experimental setup in [6]. For comparing our proposal and past works, a schema of LDM tables was applied, as mentioned in Shimada et al. [6], for their safe driving system setup. The LDM was built based on the Postgres database with PostGIS extension.

A *roadelement* table was constructed to store the lanelets corresponding to scenarios 1 and 2 static layers. In addition, an *egomotorvehicle* and *motorvehicle* layer 4 tables were built to keep the ego vehicle and non-ego vehicle information, respectively. An *alongroadelement* table was built to link the Layer 1 and Layer 4 tables ([6]).

All experiments were performed in the Ubuntu 18.04 environment on a computer with an Intel(R) Core(TM) i9-9900K CPU (3.60GHz) with 64 GB RAM. For the sake of simplicity, $a_{max}$ = 10 m/s${}^{2}$ value were assumed to friction coefficient μ=1.02 and *g* = 9.81 m/s${}^{2}$. For the generation of Kamm’s/traction circles, a time step size $\Delta t=t_{i+1}-t_{i}$ of 0.4 s and up to a time horizon of $t_{h}=1.2$ s were assumed. The BDD of all the Geohash was computed inside the concerned Kamm’s circles using Algorithm 2. Successively, the BDDs were converted to JSON format to make them suitable to save in the databases.

## 5. Results

**Figure 13:**
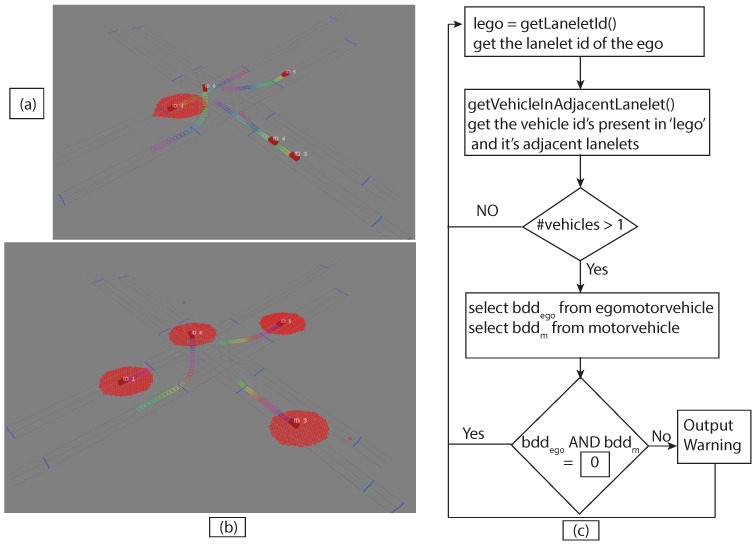
(**a**) Projected reachable Geohash for the vehicle at t∈{0.3,0.7,1.2} s. (**b**) Projected reachable Geohash for the vehicles at t∈{0.4,0.8,1.2} s. (**c**) Flow chart to avoid collision using BDD in the LDM setup.

**Figure 14:**
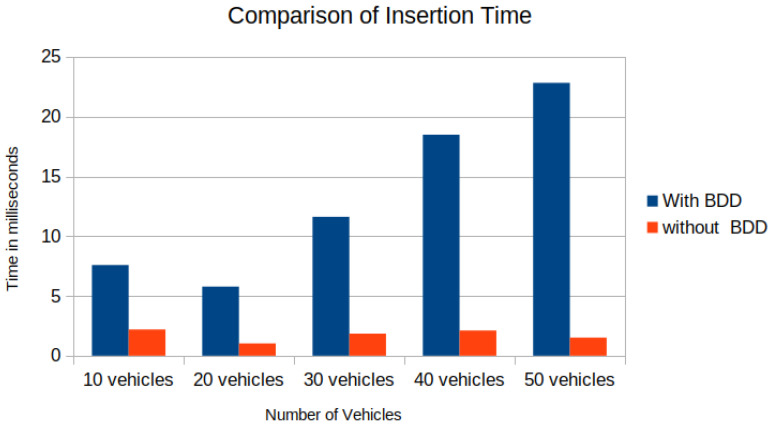
Layer 4 data insertion time with BDD vs. without BDD.

**Figure 15:**
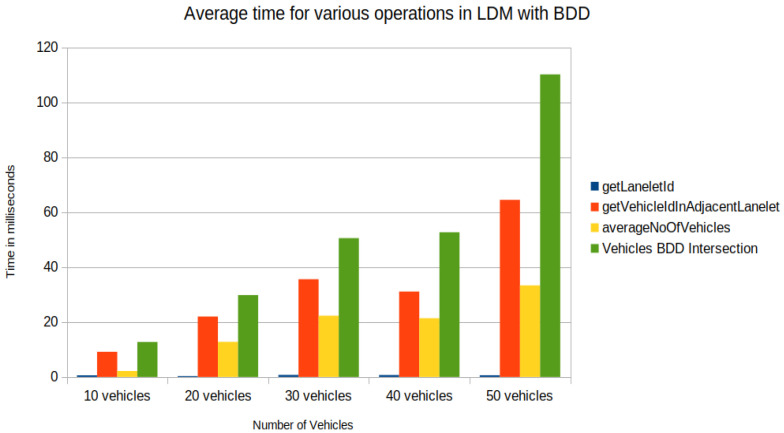
Time in milliseconds for operations (get ego vehicle lanelet id, get vehicles ids in adjacent lanelets of ego vehicle, average number of vehicles in adjacent lanelets, BDD intersection operation with adjacent vehicles for collision avoidance).

**Figure 16:**
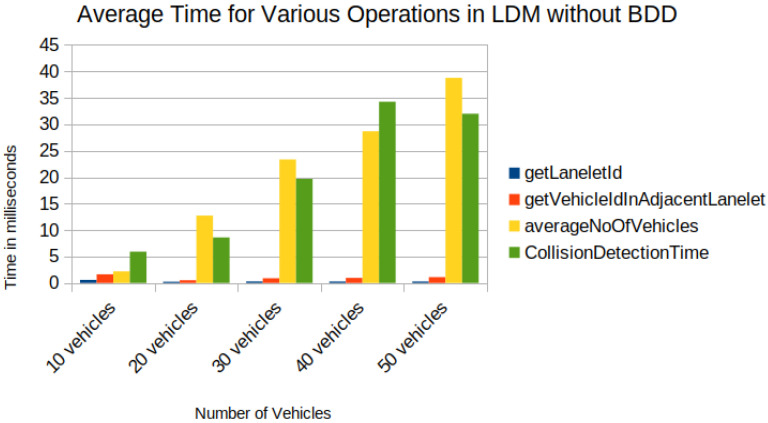
Time in milliseconds for operations (get ego vehicle lanelet id, get vehicles ids in adjacent lanelets of ego vehicle, average of vehicles in adjacent lanelets, collision risk warning algorithm from Shimada et al.).

In the experimental setup, Figure 13a shows the union of reachable Geohashes for the Kamm’s circle/reach sets at the time $t_{1}=0.3,t_{2}=0.7$ and $t_{3}=1.2$ s for the vehicle with ID = 1; Figure 13b shows the union of enclosing Geohashes at the time $t_{1}=0.4,t_{2}=0.8$ and $t_{3}=1.2$ s. In the experimental setup, the BDD was provided to represent the future occupancy for a given vehicle as $BDD_{v}=\cup_{t\in \{t_{1},\cdots ,t_{n}\}}BDD_{t}$ for Scenario 2, and we stored the data in the PostgreSQL database-based LDM in JSON format. Then, the time needed to store the vehicles layer 4 information was compared between vehicle ID, vehicle type, velocity along *x*-axis, velocity along *y*-axis, longitude, latitude, lanelet id, and current time and vehicle ID, vehicle type, velocity along *x*-axis, velocity along *y*-axis, longitude, latitude, lanelet id, $BDD_{v}$ (in JSON format), and current time. An increase in insertion time was observed when considering the BDD information as can be seen in Figure 14. Insertion time increases because the amount of data fed into the LDM increased due to the BDD. Although there was an increase in insertion time, our computer experiment demonstrated that our formulation for LDM implementation could store much richer spatial information, and even with 50 vehicles, data insertion took around 25 ms with $BDD_{v}=\cup_{t\in \{t_{0.4},t_{0.8},t_{1.2}\}}BDD_{t}$, which is suitable for the real-time operation [37]. As shown in Figure 13c, the BDD-based future occupancy information was utilized for the collision avoidance task. Figure 15 and Figure 16 indicate the timings for the following tasks:

In Figure 15 and Figure 16, the difference was observed by introducing BDD in the LDM for the following tasks:

- *Task1*—getLaneletId (to get the lanelet id and data corresponding to an ego vehicle).
- Task2—getVehicleInAdjacentLanelet (to retrieve data of all vehicles (other than ego) present in the ego vehicle’s current lanelet or its adjacent lanelets).
- *Task3*—averageNoOfVehicles (to retrieve the number of vehicles present around an ego vehicle for a given scenario).
- *Task4*—Collision avoidance, retrieve BDDs using Task2 and check for collision avoidance following the “AND" operation on BDDs in Figure 15 and collision risk warning task following the procedure in [6] for the experiment shown in Figure 16.

An increase in time for tasks was observed, such as Task2 and Task4, by introducing BDD in the LDM, as seen in Figure 15, compared to Shimada et al. [6] implementation in Figure 16. Although an increase in computation time, the proposed framework enabled the storage of possible future locations in the LDM, which is lacking in previous implementations of the LDM. Future occupation information is crucial compared to the vehicle’s current location because vehicles in middle- and long-distance ranges may interact in the future. In the current version of the implementation, nearby present vehicles may not interact in some cases. In this sense, the accuracy of collision detection has to be verified in algebraic operations over BDDs, especially around the borders of Geohash spatial segments. For up to 40 vehicles, the functions performed took less than 100 ms (Figure 15), which is applicable for real-time operation.

## 6. Discussions

In our proposal, we focused on the LDM implementation relying on V2V and V2I communications and maximized the necessary computation time in the database scheme, verified in computer experiments. On the other hand, actual vehicle verifications were out of range in the present study. The transmission delay in V2V and V2I communications is an inevitable drawback of this approach, and a sensor-based clarification of relationships with nearby vehicles is required to compensate for the drawback. As Jo et al. [38] and Vargas et al. in [3] reviewed, multiple sensor types are applicable for risk management of AVs, such as stereo cameras, light detection and ranging (LiDAR), and radars. The effective range of distance to detect obstacles varies depending on the types of sensors. According to a review [3,38], stereo cameras work from 0.5 to 3 m (Roboception) and have a limitation of 20 m (Intel RealSense). LiDAR can finely visualize targets surrounding 360 degrees within 20 m and works well in the 100 m range according to their specification. In radar, in the long-range mode, it works until 250 m, while the discrimination of targets is not assured in comparison with stereo cameras and LiDAR. The limitation of sensing range is a drawback of sensors and a fine detection of objects behind obstacles is a hard problem. On the other hand, V2V and V2I approaches as alternative solutions with respect to drawbacks of sensors also have other drawbacks, such as a transmission delay in communication among vehicles and infrastructures. An assurance of detection of non-vehicle entities as vulnerable road users such as pedestrians, joggers, and animals is highly important for safe mobility. It is possible to detect those vulnerable entities if fine sensors are embedded in road infrastructures. The coverage of sensing areas by road infrastructures is still an unsolved problem. In this sense, the sensor-based approach and V2V and V2I communications are not alternative options, which will be expected to integrate as a fusion system to guarantee road safety. Sensors are necessary for high-speed detection in the short- and middle-range of distance, which allows vehicles to avoid risks. In the middle- and long-range distances, mapping geographical information is beneficial. Our proposal extends the possibility of information sharing for vehicle future geographical occupancy information and other types of road information by using the BDD scheme. Sharing BDD-based geographical information can support to transfer of multiple road information by using algebraic operations between the exchanged BDDs. It will be crucial for risk avoidance in future interactions, which is expected with the C-ITS nature of data sharing.

In consideration of the accuracy of the future occupancy, the discrete model of Kamm’s circle was introduced and verified in the present study. Theoretically, a continuous model of Kamm’s circle is possible, while the formulation is unassured in complex traffic scenarios for safe navigation.

In consideration of improvement of computation time, LDM implementation in our results stores the reachable location up to the next 1.2 s. This factor will be improved in future deployments by storing reachable areas for a more reasonable time, in the sense of minimum swerving time for a given vehicle to avoid a collision. Furthermore, using graph-based databases may improve the latency involved due to the databases. In addition, ITE-based BDD implementation and variants of BDDs such as ZDD/MTBDD may be helpful to future potentials for improvement of the performance and functionalities of LDM. Rich algebraic properties from different decision diagrams (e.g., ZDD) can provide a solution for a new set of problems in AVs.

## 7. Conclusions

In the present study, we proposed an advanced data representation method that enables embedding future geographical occupancy of vehicles into the database using BDD. In the proposed method, future geographical occupancy of vehicles was formulated with Kamm’s circle. In computer experiments, sharing BDD-based occupancy information was successfully demonstrated in the ROS-based simulator with the linked list-based BDD on PostgreSQL as a database-based LDM, which is consistent with the C-ITS nature of data sharing. Algebraic operations between the exchanged BDDs effectively updated the possibility of future interactions, which was maintained by data insertion and timing of collision avoidance in the LDM. This result opened a new door for realizing the ideal LDM for safety in AVs.

## Figures and Tables

**Table 1:** Geographical size of Geohash encoding.

#Label in Geohash	Distance in North and South (m)	Distance in East and West (m)	A Geohash Example
1	4,989,600	4,050,000	w
2	623,700	1,012,500	wy
3	155,925	126,562.5	wyh
4	19,490.625	31,640.625	wyhb
5	4872.65625	3955.07813	wyhby
6	609.082031	988.769531	wyhby3
7	152.270508	123.596191	wyhby3k
8	19.0338135	30.8990479	wyhby3kf
9	4.75845337	3.86238098	wyhby3kf5
10	0.59480667	0.96559525	wyhby3kf5f
11	0.14870167	0.12069941	wyhby3kf5fs
12	0.01858771 (≈ 1.86 (cm))	0.03017485 (≈ 3.02 [cm])	wyhby3kf5fst

## Data Availability

The map used in this article is Bing imagery available at https://www.bing.com/maps/ (accessed on 9 January 2022) and was edited using JOSM as cited in Java OpenStreetMap editor [36].
